# Estimated collective effective dose to the population from radiological examinations in Slovenia

**DOI:** 10.2478/raon-2014-0028

**Published:** 2015-03-03

**Authors:** Dejan Zontar, Urban Zdesar, Dimitrij Kuhelj, Dean Pekarovic, Damijan Skrk

**Affiliations:** 1 Slovenian Radiation Protection Administration, Ljubljana, Slovenia; 2 Institute of Occupational Safety, Ljubljana, Slovenia; 3 Clinical Radiology Institute, University Medical Center Ljubljana, Ljubljana, Slovenia

**Keywords:** collective effective dose, population exposure, dose per capita, radiological procedures, diagnostic procedures

## Abstract

**Background:**

The aim of the study was to systematically evaluate population exposure from diagnostic and interventional radiological procedures in Slovenia.

**Methods:**

The study was conducted in scope of the “Dose Datamed 2” project. A standard methodology based on 20 selected radiological procedures was adopted. Frequencies of the procedures were determined via questionnaires that were sent to all providers of radiological procedures while data about patient exposure per procedure were collected from existing databases. Collective effective dose to the population and effective dose per capita were estimated from the collected data (DLP for CT, MGD for mammography and DAP for other procedures) using dose conversion factors.

**Results:**

The total collective effective dose to the population from radiological in 2011 was estimated to 1300 manSv and an effective dose per capita to 0.6 mSv of which approximately 2/3 are due to CT procedures.

**Conclusions:**

The first systematic study of population exposure to ionising radiation from radiological procedures in Slovenia was performed. The results show that the exposure in Slovenia is under the European average. It confirmed large contributions of computed tomography and interventional procedures, identifying them as the areas that deserve special attention when it comes to justification and optimisation.

## Introduction

Medical procedures using ionizing radiation represent by far the largest source of manmade population exposure to ionizing radiation in most developed countries. In the USA the effective dose from medical exposure already exceeded the contribution from the natural background.^1^ As exposure is related to cancer risk^2^ it is important to determine the contribution of doses from medical exposure to the population as required by the legislation of European Union.^3^,^4^ While numerous studies were carried out in the past^1^,^5^–^11^ and the results from European countries^5^ show that in Europe contribution of medical procedures to the population exposure is significantly lower than in the USA^2^, no systematic study has been previously performed in Slovenia.

This article reports the findings of the first systematic study, carried out in 2011 by the Slovenian Radiation Protection Administration (SRPA) in scope of the European Commission project “Study on European Population Doses from Medical Exposure” or “Dose Datamed 2”. A standard methodology^5^ developed by the first “Dose Datamed” project and recommended by the European Commission was adopted.

## Materials and methods

A precise determination of population exposure from medical use of radiation would require detailed knowledge of number of procedures and patient doses for all procedures. As full information is not realistically obtainable, three models for data collection were proposed by the Dose DataMed 2 project. They were based on TOP20 types of examination, 70 examination categories or 225 specific examinations.^5^ Based on the available informational infrastructure the TOP20 approach was chosen for Slovenia. It is based on collecting frequency and dose information for 20 examination types as listed in guidance RP154^5^ that were determined to present major contribution to the total population exposure. They consist of 7 examination types belonging to conventional radiography (without contrast media), 5 radiography/fluoroscopy examination types (mostly involving contrast), 7 categories of computed tomography examinations and coronary angioplasty (PTCA) as a representative of interventional procedures. All 20 examination types are listed in the first column of Table 1. The results obtained by the TOP20 methodology were extrapolated to the overall collective effective dose using correction factors proposed by the Dose Datamed 2 project. To determine the effective dose per capita the overall collective effective dose was divided by the total population of Slovenia at the end of 2011 *i.e.* 2.05 million.^12^

### Frequency estimation

Data on the frequencies of the selected procedures were collected using a questionnaire based on the RP154^5^ guidance. In order to minimise an error originating from different classification of the procedures appropriate instructions were included. For each of the 20 types of examination information on typical examinations, common techniques and common indications were provided. A single procedure was defined as one examination that may consist of more projections or, in case of CT, phases. In case of mammography both projections on both breasts were defined as one examination. For examinations that may consist of more projections, frequencies were requested for all projections. For CT procedures the average number of phases used for each type of examination was required.

Review of SRPA databases identified 90 providers that can perform those types of examinations. The questionnaire was sent to all of them and the response rate was 80% (72 providers), covering approximately 90% of the total workload. Only two of the major institutions (General hospitals Brežice and Murska Sobota) did not provide the data so the frequencies had to be estimated. For CT procedures, health insurance agency data for the year 2009 were available and adopted. For other procedures, frequencies were estimated from the CT frequencies by using an average ratio between CT and other procedures as determined for the general hospitals that reported full data. For the remaining providers the frequencies were estimated from the (generally conservative) workload information as obtained during licensing procedures. The influence of those estimations on the cumulative dose will be discussed in the section dedicated to the uncertainty of the results.

### Dose estimation

The average effective dose for each examination type was determined from measurements of the relevant dose quantities on a sample of patients during regular practice. Dose Area Product (DAP) was used for conventional, fluoroscopic and interventional procedures, Dose Length product (DLP) for CT procedures and Mean Glandular Dose (MGD) for mammography. The measurements were performed by the Institute of Occupational Safety during the previous years and were available for approximately 2/3 of institutions performing conventional radiography, for about 80% of CT units, for all mammography units (MGD from phantom measurements) and for all units performing PTCA (cumulative DAP) and cardiac angiography. Data were very scarce (available only for one to four providers per procedure) for the remaining fluoroscopy procedures (DAP per procedure). For examinations that may consist of more projections, the average cumulative quantity was determined by summing the relevant values for all projections weighted by the fraction of the examinations for which each projection was carried out. For example, in case of chest x-ray total DAP would be determined as DAP_chest_ = DAP_PA_ + F_LAT_^*^DAP_LAT_, where DAP_PA_ and DAP_LAT_ are average DAP values for PA and lateral projection and F_LAT_ is the fraction of all examinations where imaging is also performed in lateral projection. For CT examinations the average DLP per phase was multiplied by the average number of phases used for a given type of examination. Average values of the relevant dosimetric quantities for each type of examination were then converted to the average effective dose per procedure using conversion coefficients recommended by the guidance RP154^5^, except for mammography where conversion coefficient of 0.12 mSv/mGy was used following ICRP publication 103.^4^ Considering that mammography is mostly performed on older patients conversion coefficient of 0.05 mSv/mGy as recommended by the ICRP publication 60 could be used instead.^13^ In that case the total dose from mammography would be reduced by a factor 2.4 and the overall cumulative dose by approximately 2%. Values of the conversion coefficients for all 20 examination types as used in this research are listed in Table 2.

Collective effective dose D_col, ex_ for each examination was calculated as a sum of the frequencies of that examination type in each institution N_ex,inst_ multiplied by the effective dose for that examination in the corresponding institution D_ex_, _inst_ i.e. D_col, ex_ = ∑_inst_ N_ex_,_ inst_ ·D_ex, inst_.

For institutions for which an effective dose for any given type of examination was not known the average effective dose for that type of examination over all institutions was used instead. The total collective effective dose for the TOP20 procedures was determined as a sum of collective effective doses over all 20 examinations.

## Results

### Frequency data

Frequency data for all 20 procedures as collected by this study are shown in Table 1. For the purpose of this article the radiology providers were categorised into 5 groups: university medical centres (2), general hospitals (10), specialised hospitals and larger practices (13), public health centres (38) and other institutions including mostly smaller private practices (27). As it was not possible to present full data such grouping was considered as a useful compromise that still provides some insight into contributions of different types of providers. For each group the frequencies are reported for each type of procedure and summed up for each group of procedures. Total number of procedures for each type of examination is given in the seventh column of Table 1. In the last column some basic information about the procedures that influence the patient exposure are listed: in case of conventional radiography the fraction of examinations for which lateral projection is used (except for mammography where both projections on both breasts are assumed for all examinations), for radiography/fluoroscopy number of images per examination and for CT procedures the average number of phases used per examination. In all cases the average value over all institutions, not weighted by their workload, is listed.

The results show that in 2011 nearly 1 million of the TOP20 types of examinations were performed in Slovenia. Approximately 88% of them belong to conventional radiography, approximately 10% were CT procedures, radiography/fluoroscopy examinations contributed about 1.5% and interventional procedures approximately 0.5%.

Data obtained by the TOP20 methodology can be used to estimate the overall number of radiological procedures (including dental) using correction factors as determined by the Dose Datamed 2 project.^14^ They were determined from results of the countries that collected data based on both TOP20 types of examination and 225 specific examinations. The values of the correction factors and the resulting estimates for the overall number of radiological procedures are listed in Table 2. The extrapolation of data shows that about 2 millions of radiological procedures were performed in Slovenia in 2011, corresponding to one procedure per capita. The relative contributions of each group of procedures are slightly modified when looking at the overall numbers instead of the TOP20 procedures with the contribution of conventional radiography being increased to about 92% and contribution of CT procedures reduced to about 5%. Contributions of radiography/fluoroscopy and interventional procedures remain at almost the same values of around 1.5% and 0.5% respectively.

### Dose data

To determine the cumulative dose the frequency data were combined with the dose information as described in the section about methodology of the study. The average values of the relevant dose quantities *i.e.* DAP per projection for conventional radiography, DAP per examination for radiography/fluoroscopy and interventional procedures, DLP per phase for CT procedures and MGD per breast (both projections) for mammography are listed in Table 3. For the first five procedures from the conventional radiography group separate DAP values were used for AP/PA and lateral projections so average values are listed for both projections. In the analysis the DAP value for each lateral projection was properly weighted with the frequency of examinations in which both projections were taken (overall averages are listed in the last column of Table 1). For mammography, the listed values were multiplied by 2 to obtain MGD per examination *i.e.* imaging of both breasts. For CT examinations DLP per phase was multiplied by the average number of phases (overall averages in Table 1) to obtain DLP per examination as defined by the Dose Datamed 2.

The third column in Table 3 lists the conversion coefficients for all TOP20 examinations as used in this study and in the fourth column cumulative effective doses for all TOP20 types of procedures and for each of group of procedures are given in units of man·Sv. The last column lists the average effective dose per examination for all TOP20 types of examinations. They were determined by dividing the cumulative effective dose from each type of examination by the corresponding number of examinations and thus represent averages weighted by the workload of each institution. While not directly relevant for the evaluation of collective dose of the general population they offer a good insight into both absolute and relative dose burden of a patient subjected to radiology examinations in Slovenia.

As it was done for the frequency data, cumulative dose from the TOP20 procedures can be extrapolated to estimate an overall cumulative dose using correction factors as determined by the Dose Datamed 2 project.^14^ The correction factors for dose and the resulting overall cumulative doses for each group of procedures are given in Table 4. The extrapolated data indicate that the overall dose to the population of Slovenia from radiological procedures in 2011 was about 1300 manSv or 0.65 mSv per capita. The main contribution, almost 2/3, is due to computed tomography, conventional radiography contributes approximately 20%, interventional procedures around 10% and radiography/ fluoroscopy only about 5%.

### Uncertainties of the results

Three main contributions to the uncertainty of the overall cumulative effective dose are: uncertainty on the frequencies of procedures, uncertainty in dose estimation and uncertainty of the extrapolation to the overall cumulative dose from the TOP20 data.

Frequency data as reported in the survey were mostly extracted from databases of each provider. Depending on the available technology either exact numbers were extracted from an electronic system or yearly workload was extrapolated from a shorter (usually a few months) time period. Another source of frequency uncertainty could be procedure mismatching. A 10% uncertainty was conservatively assumed for institutions that reported frequency data. For the institutions for which frequencies had to be estimated from other sources a 50% uncertainty was assumed. Uncertainties for each institution and for each type of examinations were assumed to be uncorrelated and absolute values were summed in quadrature. Using this methodology a 2% uncertainty on the frequencies of the TOP20 examinations was estimated for conventional radiography, around 4% uncertainty for radiography/fluoroscopy procedures, around 3% uncertainty for CT procedures and approximately 6% uncertainty for interventional procedures. Relative uncertainty on the total number of the TOP20 radiological procedures was around 2%.

As reported in a previous section, values of the relevant dose quantities were known for most institutions from previous studies. For majority of the examinations the effect of the uncertainty on dose per procedure was estimated by substituting individual matching of dose data and frequencies for each institution with using the average dose value of each type of examination for Slovenia for all institutions. Such approach gave approximately 5% uncertainty on cumulative dose from conventional radiography, 3% for CT procedures and 3% for interventional procedures. For the four radiography/fluoroscopy examinations (excluding coronary angiography) where the data were very scarce a 100% uncertainty on the dose was assumed, leading to a 25% uncertainty on the cumulative dose from radiography/fluoroscopy examinations (including coronary angiography). Combining all those uncertainties in quadrature gives less than 3% overall uncertainty due to dose data. Uncertainty on the dose conversion coefficients was ignored for the purpose of this study.

To estimate the uncertainty on the overall cumulative effective dose from radiological procedures uncertainties on frequency and dose estimations for the TOP20 procedures had to be combined with the uncertainty of the correction factors used to extrapolate the TOP20 data to all examinations. The values of those correction factors as proposed by the Dose Datamed 2 project were given in Table 4. Unfortunately the Dose Datamed 2 report^14^ provided no uncertainty values for those factors. The uncertainty on the conversion factors was thus estimated from the data available in the report to around 25% for conventional radiography group, 40% for radiography/fluoroscopy group, 13% for CT group and 20% for interventional procedures group. It can be seen that the total uncertainty on the overall effective dose from radiography procedures in Slovenia is dominated by the uncertainty of the correction factors. Combining all three main sources of uncertainty for each group of examinations separately and then combining data from all four groups of examinations, assuming the uncertainties were uncorrelated, the total uncertainty on collective effective dose from radiology procedures was estimated to be around 11% (1 standard deviation).

## Discussion

The results presented above provide an estimate of the collective effective dose to the population of Slovenia from radiological examinations. They show that computed tomography, while only representing about 5% of all radiology procedures in Slovenia, contributes approximately 2/3 of the total dose and is thus is a major source of exposure to the population. CT was therefore identified as the main area towards which further efforts for increasing optimisation and justification should be directed. Another area that deserves special attention are interventional procedures that only represent around 0.5% of all radiology procedures but contribute approximately 10% to the overall cumulative effective dose. While those conclusions could be expected based on the previous studies from other developed countries this is the first time that reliable information are available about the situation in Slovenia.

The study shows that in conventional radiography workload is about equally distributed between public health centres and other small providers (approximately 1/3), general hospitals and university medical centres (another third) and specialised hospitals and larger providers (the last third). Among CT examinations approximately 30% are performed in the university medical centres, 45% in general hospitals and the remaining 25% in specialised hospitals and larger private centres with the later contributing approximately 10%. As for the interventional procedures, approximately 80% are performed in the university medical centres and the remaining 20% in 2 general hospitals and one private centre. The above sharing is expected to be somewhat modified if the TOP20 list is expanded to all procedures, particularly if dental radiography is included in conventional radiography.

The presented study was conducted according to a well defined and internationally accepted methodology. Such approach provided a well developed methodology and ensured that the results can be reliably compared with other European countries.^5^,^14^ The comparison shows that the overall effective collective dose per capita in Slovenia is below average for European countries (around 1.1 mSv per capita^14^ for 2011) and places Slovenia among the countries with the lowest overall effective collective dose per capita. Comparison of the overall total frequencies per 1000 population^14^ places Slovenia into the middle of distribution with 20 countries having higher and 15 countries lower overall total frequencies. The relative frequency of computed tomography in Slovenia (5%) tends to be lower than in many countries (only 8 out of 36 countries evaluated in the Dose Datamed 2 report^14^ show lower relative frequency of CT procedures than Slovenia with only two of them lower than 4%). This indicates either good use of the principle of justification when referring to CT examinations or somewhat limited access to this modality caused by lower number of CT units per capita than the European average.^15^ On the other hand the relative contribution of CT procedures to the overall effective dose (63.8%) is higher from the reported mean value (57%) for all countries. This is consistent with the observation that the average doses for most CT examinations from the TOP20 group in Slovenia significantly exceed the mean over the countries included in the study. It is thus necessary to put more efforts into optimisation of CT procedures in Slovenia. On the other hand the average doses for conventional radiography in Slovenia are significantly lower from the mean values as given in the Dose Datamed 2 report. Thus the relative contribution of conventional procedures to the cumulative effective dose (19.3%) is comparable to the reported mean value (19.5%) while the relative frequency (92.8%) is above the reported mean of 87.4%. Interventional procedures have comparable contribution to the cumulative effective dose (0.6%) to the mean (0.6%) despite slightly larger relative frequency (11% *vs.* 8.7%). The relative contribution of the radiography/fluoroscopy group in Slovenia is lower from the mean both in frequency (1.5% *vs.* 3.3%) and in cumulative dose (6.0% *vs.* 14.8%).

Although it was not the main goal of the study the extensive data collection on which it was based provided a wealth of other information about the radiology practice in Slovenia. An example is information about the relative frequency of using lateral projection in chest imaging. The data show that both PA and lateral projection are in average taken in 45% of all chest x-rays. If we take under investigation public health centres where similar clinical questions can be assumed it can be seen that in approximately 1/3 of all public health centres both projections are performed in over 80% of all chest x-rays while in about 1/3 of them lateral projection is taken in less than 20% of all chest x-rays. Another side result of the study is information about the average patient doses for the TOP20 procedures in Slovenia (Table 3 last column). While not relevant for optimisation purposes the values are still indicative for evaluation of the relative risk of different procedures and could be useful for educational purposes. The authors are aware that the full potential of the data collected in this study data was not yet explored. Such analysis exceeds the scope of this article and may become a topic of a separate study.

## Conclusions

Results of the first systematic study of population exposure in Slovenia due to radiological medical procedures are presented. They show that total collective effective dose from radiological procedures in 2011 was approximately 1300 manSv. By far the largest share is due to computed tomography that contributes approximately 830 manSv or almost 2/3 of the total dose, although it only represents approximately 5% of all diagnostic procedures. Another important group are interventional procedures that represent approximately 0.5% of the total workload but contribute approximately 10% of the cumulative dose. Those two groups were thus identified as the areas that deserve special attention when it comes to justification and optimisation. Results of the study on nuclear medicine that was reported in a previous article^16^ showed that the total collective effective dose from nuclear medicine procedures in 2011 was approximately 100 manSv or 0.05 mSv per capita. Adding the nuclear medicine contributions the overall collective effective dose from medical examinations in Slovenia in 2011 was approximately 1400×(1±0,1) manSv or 0.7×(1±0,1) mSv per capita.

Presented results show that population exposure from medical procedures in Slovenia is in most aspects comparable to, or even lower than in most European countries. The one exception is computed tomography that represents much lover fraction of the total frequency yet still has a relatively high contribution to the cumulative dose.

Due to the rapid technological development and ever-increasing utilisation of radiological examinations, particularly the high-dose procedures such as CT, surveys and analysis of the doses from medical procedures should be performed regularly. The results of the presented study provided reliable information about the contribution of various types of radiological examinations to the population exposure, contributions of various radiology providers as well as some insight into the differences in everyday practice among them. This information can and should be used to direct the efforts of radiology specialists and regulators to the most critical areas while regularly updated information would provide insight into the impact of the changing technologies and guidelines.

## Figures and Tables

**TABLE 1.** Number of radiology procedures performed in 2011 for the selected 20 types of procedures

**Type of examination**	**University medical centres (2) [×1000]**	**General hospitals (10) [×1000]**	**Other hospitals (13) [×1000]**	**Public health centres (38) [×1000]**	**Other institutions (27) [×1000]**	**Total (90) [×1000]**	**Average no. of ...... per examination**
**Conventional radiography**	**196**	**279**	**120**	**226**	**45**	**865**	**Side proj.**
Chest/lung	109	116	47	100	27	399	0.45
Cervical spine	17	37	6.2	23	2.7	86	0.91
Thoracic spine	10	14	3.1	10	1.2	39	0.94
Lumbar spine	24	39	12	43	3.3	121	0.87
Mammography	0,6	27	39	28	8.8	104	
Abdomen	11	25	2.5	0,6	0.1	39	0.17
Pelvis & hip	24	22	9.8	21	2.1	79	0.26
**Radiography/ fluoroscopy**	**8.3**	**4.2**	**2.5**	**<0,1**	**0**	**15**	**Images**
Ba meal	0.3	0.8	1.0	<0.1	0	2.1	4.93
Ba enema	0.5	0.8	0.1	0	0	1.4	5.26
Ba follow	0.3	0.2	0.6	0	0	1.1	5.25
Intravenous urography (IVU)	1.1	1.4	0.1	0	0	2.6	4.58
Cardiac angiography	6.2	0.9	0.7	0	0	7.8	
**Computed tomography**	**27**	**44**	**24**	**0**	**0**	**96**	**Phases**
CT head	16	25	8.7	0	0	49	1.57
CT neck	1.2	1.0	0.7	0	0	2.9	1.13
CT chest	3.2	4.1	5.9	0	0	13	1.47
CT spine	0.5	2.6	3.3	0	0	6.4	1.00
CT abdomen	4.7	9.2	3.9	0	0	18	2.24
CT pelvis	0.2	0.6	0.3	0	0	1.1	1.48
CT trunk	1.4	2.1	1.5	0	0	5.1	2.00
**Interventional procedures**	**3.2**	**0.5**	**0.2**	**0**	**0**	**3.9**	
PTCA	3.2	0.5	0.2	0	0	3.9	
**Total**	**234**	**328**	**147**	**226**	**45**	**980**	

**TABLE 2.** Estimated total number of radiological procedures performed in Slovenia in 2011

**Type of examination**	**Number of TOP20 examinations [×1000]**	**Correction factor**	**No. of overall examinations [×1000]**	**Relative contribution to overall number [%]**
Conventional radiography	865	2.25	1947	92.8
Radiography/ fluoroscopy	15	2.04	31	1.5
Computed tomography	96	1.13	108	5.1
Interventional procedures	3.9	3.23	12	0.6
**Total**	**980**		**2098**	

**TABLE 3.** Summary of the dose information for the selected 20 types of procedures

**Type of examination**	**Average value of the relevant dose quantity CT: DLP [mGycm] Mamo: MGD [mGy] Other: DAP [μGym2]**		**Conversion coefficient CT: Sv/Gycm Mamo: Sv/Gy Other: Sv/Gym2**	**Collective effective dose [manSv]**	**Average effective dose per procedure [mSv]**
**Conventional radiography**	**PA/AP**	**LAT**		**225**	
Chest/lung	11.0	33.3	1.80	18.2	0.05
Cervical spine	24.5	23.1	1.30	5.5	0.06
Thoracic spine	101	93	1.90	13.8	0.36
Lumbar spine	124	219	2.10	91.9	0.76
Mammography	1.5		0.12	38.7	0.37
Abdomen	170		2.60	16.2	0.42
Pelvis & hip	197		2.90	40.2	0.51
**Radiography/fluoroscopy**				**55.6**	
Ba meal	700		2.00	2.9	1.4
Ba enema	2800		2.80	11.2	7.8
Ba follow	2400		2.20	5.8	5.3
Intravenous urography (IVU)	483		1.80	2.4	0.9
Cardiac angiography	2110		2.00	33.4	4.3
**Computed tomography**				**675**	
CT head	875		2.10	142	2.9
CT neck	580		5.90	8.6	3.0
CT chest	385		14.0	88.7	6.7
CT spine	650		15.0	64.0	9.9
CT abdomen	467		15.0	273	15.3
CT pelvis	415		15.0	10.7	9.8
CT trunk	852		15.0	88.8	17.5
**Interventional procedures**					
PTCA	6000		2.00	47.8	12.4
**Total**				**1004**	

**TABLE 4.** Cumulative dose from radiological procedures extrapolated to all examinations

**Type of examination**	**Collective effective dose from TOP20 methodology [manSv]**	**Correction coefficient for extrapolation to all examinations**	**Extrapolated collective effective dose to all examinations [manSv]**	**Contribution to total collective effective dose [%]**
**Conventional radiography**	225	1.12	252	19
**Radiography/fluoroscopy**	55.6	1.40	78	6
**Computed tomography**	675	1.23	831	64
**Interventional procedures**	47.8	2.97	142	11
**Total**	**1004**		**1302**	
